# Interluekin-35 in Asthma and Its Potential as an Effective Therapeutic Agent

**DOI:** 10.1155/2017/5931865

**Published:** 2017-04-30

**Authors:** Peng Gao, Zhenzhong Su, Xuejiao Lv, Jie Zhang

**Affiliations:** Department of Respiratory Medicine, The Second Affiliated Hospital of Jilin University, Changchun, China

## Abstract

Interleukin- (IL-) 35 is a member of the IL-12 cytokine family and a heterodimeric protein formed by Epstein-Barr-induced gene 3 (EBI3) and IL-12p35. Emerging evidence shows that IL-35 is a key player in the regulation of cellular communication, differentiation, and inflammation. Altered IL-35 expression has been found in disease conditions such as cancer, rheumatoid arthritis, and, more recently, asthma. In cancer, IL-35 is involved in the regulation of tumorigenesis, cancer progression, and metastasis. In rheumatoid arthritis, IL-35 acts as a negative regulator of inflammation. Similarly, IL-35 also appears to suppress allergic inflammation in asthma. In an in vivo murine model of asthma, transfer of adenovirus-mediated IL-35 markedly reduced the degree of airway hyperresponsiveness (AHR) and inflammatory cell infiltration. Many studies have shown the involvement of IL-35 in a number of aspects of allergic inflammation, such as eosinophil and neutrophil recruitment as well as inhibition of inflammatory mediators of the Th2 subtype. However, the exact molecular mechanisms underlying the role of IL-35 in human asthma have yet to be fully elucidated. This review describes the current evidence regarding the role of IL-35 in the pathophysiology of asthma and evaluates the potential of IL-35 as a biomarker for airway inflammation and a therapeutic target for the treatment of asthma.

## 1. Introduction

Interleukin- (IL-) 35 is a member of the IL-12 cytokine family [1, 2] and was first described almost two decades ago as a heterodimer formed by the subunits Epstein-Barr-induced gene 3 (EBI3) and IL-12p35. It was found in the trophoblast component of the human placenta and suggested to be an important immunoregulator already upon discovery [3]. IL-35 induces the generation of a unique class of IL-35-producing regulatory T cells (Tregs) that are distinctly different from the other transforming growth factor beta- (TGF-*β*-) or IL-10-induced Tregs. These cells are called IL-35-producing iTregs (iTr35 cells) [1, 4]. IL-35 secreted by iTr35 cells in turn induces the generation of even more iTr35 cells, and thus, IL-35 and iTr35 cells create a positive feedback loop together [1].

**Figure 1:**
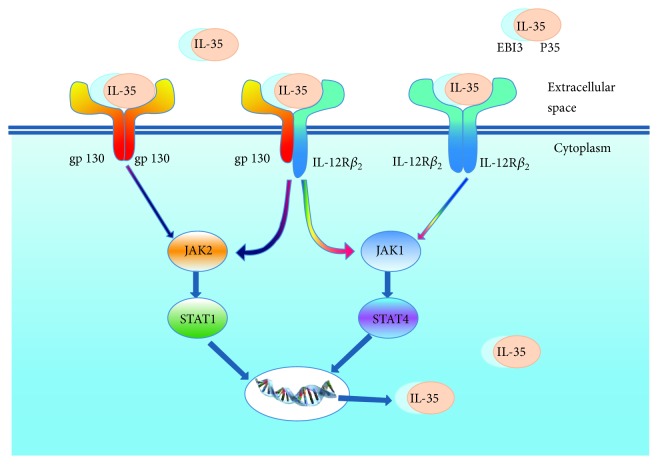
The structure of IL-35 and the activation of the JAK-STAT signaling pathway.

The IL-35 receptors on the cell surface can be either homodimers of IL-12R*β*_2_ or gp130 or a heterodimer of IL-12R*β*_2_:gp130 [2]. Binding of IL-35 to its receptors leads to activation of the JAK-STAT pathway, which requires the transcription factors STAT1 and STAT4 to form a unique heterodimer [2] and induces immunosuppression, IL-35 production, and conversion of naive T cells into iTr35 cells. The heterodimeric IL-12R*β*_2_:gp130 receptor is required for the maximal effects to be achieved [2] (Figure 1). While the gp130 subunit is widely expressed in many different cell types, the IL-12R*β*_2_ subunit is mainly found on activated T cells of the Th1 subtype and natural killer cells [5]. In most resting T cells, IL-12R*β*_2_ is almost undetectable. However, exposure to IL-2, IL-12, IL-27, interferon- (IFN-) *γ*, and tumor-necrosis factor (TNF) can rapidly increase the expression of IL-12R*β*_2_ [2]. Expression of IL-12R*β*_2_ has also been shown on other immune cells, such as dendritic cells [6].

IL-35 is produced by a wide range of tissues and cell types, including monocytes, T cells, B cells, Tregs, and tumor cells under resting conditions [7, 8]. Upon Toll-like receptor stimulation, IL-35 subunits are also produced by macrophages and dendritic cells [9]. Recently, Guttek and Reinhold showed that both resting and stimulated CD4^+^CD25^+^ T cells from humans secrete high amounts of IL-35, whereas peripheral pan T cells or CD4^+^, CD8^+^, and CD4^+^CD25^−^ T cells produce IL-35 only upon stimulation [10].

The primary physiological role of IL-35 is to regulate T cell homeostasis. It inhibits T helper (Th) 2 and Th17 polarization. Niedbala et al. [7] demonstrated that IL-35 inhibits Th17 cell differentiation in vitro and suppresses IL-17 production in vivo in mice. Similar conclusions were reached by Liu et al. [11] who demonstrated increased T cell production of IL-17 in the peripheral lymphoid organs of EBI3-deficient mice, supporting an inhibitory role for EBI3 against Th17 polarization. Spleen cells from mice deficient in the IL-35 subunit EBI3 display increased expression of IL-17 and IL-22 as well as retinoid-related orphan receptor *γ*t (ROR*γ*t), which is the key transcription factor regulating Th17 cell differentiation [12]. IL-35 secreted by inducible costimulator-positive Tregs has been found to suppress Th17 activity and thereby reduce the degree of neutrophilia and airway hyperresponsiveness (AHR) found in the airways of mice sensitized and challenged with ovalbumin (OVA) [13]. IL-35 also expands Tregs, specifically iTr35 cells, both in vitro and in vivo [1, 7]. However, the results have not been unanimous, as one study also showed a reduction in the Treg response induced by IL-35 [11].

We searched PubMed for papers published between the periods 2000 and 2017 including the key word “IL-35”. Relevant papers were included after manual selection based on the abstracts and discussion within the research group. The present review summarizes the published literature on the functions and mechanisms of IL-35 in both humans and experimental models, explores its role in the pathophysiology of asthma, and discusses the potential of IL-35-based therapeutic strategies against asthma.

### 1.1. Expression of IL-35 in Human Diseases

The body of literature studying the effects of IL-35 has expanded rapidly in recent years. Such increased interest might be due to the broad range of biological functions associated with IL-35, including cell differentiation, tumor progression, and immunoregulation [1, 14, 15]. Moreover, IL-35 has been found to be involved in the physiopathology of a range of different diseases (Table 1). The reported normal levels of IL-35 are however highly variable in literature. This might be due to the different ELISA kits and standards used.

To date, many aspects of inflammatory and autoimmune disease have been linked to IL-35 function, such as suppression of CD4^+^ effector T cells [1, 7, 16, 17], Th17 cell development and differentiation [7], and attenuation of allergen-specific Th2 responses and production of Th2 cytokines [18]. Initially, IL-35 was thought to have anti-inflammatory effects on immune-mediated inflammatory diseases through the suppression of T cell proliferation and effector functions. However, a recently published study demonstrated a paradoxal proinflammatory effect of IL-35. In mice with collagen-induced arthritis (CIA), treatment with IL-35 gene transfer actually exacerbated arthritis symptoms and at the same time increased the Th17 cell/Treg ratio in the spleen [19]. These contradictory results suggest that the in vivo functions of IL-35 are complex and that additional studies are required to truly elucidate the roles of IL-35 in health and disease.

Aberrant expression of extracellular IL-35 has been detected in the inflammatory setting both in animal models and human serum in vivo. Remarkably increased expression of EBI3 and p35 was detected in intestinal epithelial cells from wild-type mice by qPCR. Intestines of EBI3-deficient mice showed increased degree of colitis compared to those of control mice, while treatment with exogenous recombinant IL-35 decreased intestinal inflammation by suppression of Th1- and Th17-dependent inflammatory responses [9]. In humans, patients with ulcerative colitis (UC) and Crohn's disease (CD) were found to have significantly lower levels of serum IL-35 than healthy individuals, and the cytokine level was inversely correlated with UC activity. The gene and protein expression levels of EBI3 and p35, which are the two subunits of IL-35, were significantly higher in the intestinal mucosa of patients with inflammatory bowel disease (IBD) compared to that of healthy controls [20]. Atherosclerosis and coronary artery disease (CAD) have long been thought to be caused by disrupted lipid metabolism but have gained wide recognition as inflammatory conditions in recent years. It has been shown that plasma IL-35 levels are significantly lower in patients with stable and unstable angina pectoris, as well as acute myocardial infarction compared with the levels in patients with chest pain syndrome. In CAD patients, IL-35 levels were negatively correlated with the degree of heart failure, defined by a decreased left ventricular ejection fraction (LVEF) [21].

### 1.2. The Role of IL-35 in Human Asthma

Asthma is a chronic inflammatory condition of the respiratory system characterized by abnormal T cell responses [29]. The asthmatic inflammation is finely orchestrated by CD4^+^ T lymphocytes, including Th1, Th2, and Th17 cells [30]. Airway inflammation leads to increased bronchial contractions termed airway hyperresponsiveness (AHR) and asthmatic symptoms such as wheezing, shortness of breath, and chest tightness [31].

Recent research has shown that asthma is a heterogeneous disease, and much effort has been put into subtyping the different phenotypes of asthma. A common method of subtyping asthma is based on the presence of different inflammatory cells in induced sputum, with a classification into four different groups: neutrophilic asthma, eosinophilic asthma, mixed granulocytic asthma, and paucigranulocytic asthma. Each subtype has a distinct mechanism and responds to therapy differentially [32–34]. Recognition that asthma is not one homogenous disease and that different subtypes of asthma exist is crucial for understanding the underlying disease processes and the development of the so called “personalized medicine.” Classic eosinophilic asthma is the best-characterized subtype and responds well to corticosteroids, which are the first-line therapy against asthma used in the clinic today. The mechanisms underlying eosinophilic asthma primarily involve allergen-induced activation of Th2 pathways and release of Th2 cytokines, such as IL-4, IL-5, IL-9, and IL-13 [35]. Neutrophilic asthma, on the other hand, is driven by the activation of the innate immune system including Toll-like receptors (TLRs) and NLRP3 inflammasome by infections and pollutants [36, 37]. This subtype of asthma is much less well characterized and notoriously resistant to corticosteroid treatment. The innate immune system including Toll-like receptors (TLRs) and NLRP3 inflammasome has been shown to be involved in neutrophilic asthma [36, 37]. More recently, Th17 cells, which produce IL-17 and IL-22 and mediate neutrophil recruitment [38], have also been shown to have an influential role [39].

**Figure 2:**
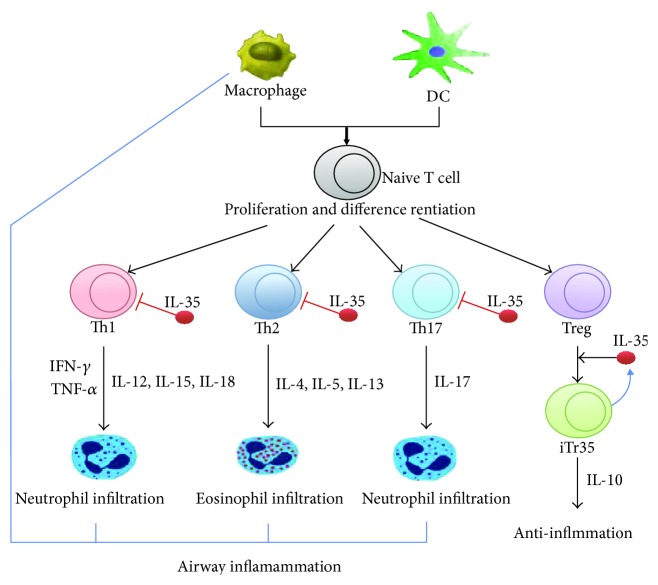
Function of IL-35 in asthma. The black arrow indicates positive effects, and the T-shaped end indicates negative effects. DC: dendritic cell; IL: interleukin; Th: helper T cell. Adapted from Pope and Shahrara [4].

IL-35 may be differentially involved in the pathogenesis of the different phenotypes of asthma. IL-17, which can be suppressed by IL-35, is involved in both neutrophilic and eosinophilic asthma. IL-17 produced by Th17 cells or macrophages not only promotes the infiltration of neutrophils into the lung but also, as suggested by recent evidence, can contribute to the development of allergic eosinophilic asthma [40, 41] and act synergistically with Th2 cytokines to promote AHR [39, 40, 42]. Tregs can secrete IL-35 [3], which in turn suppresses Th1, Th2, and Th17 effector responses to keep inflammatory responses in check [43, 44] (Figure 2). Defective Treg regulation is associated with the pathogenesis of both Th2- and Th1-mediated inflammatory diseases [45, 46], including asthma, which is a classic Th2-mediated inflammatory disease [47, 48]. Interestingly, decreased numbers of Tregs were also detected in the lungs of patients with tobacco smoking-induced emphysema [49], suggesting that Tregs regulate not only asthmatic inflammation but also emphysema-related inflammation in the lungs. Rats exposed to cigarette smoke also had significantly lower IL-35 concentrations in their bronchoalveolar lavage fluid (BALF) compared to control rats [50].

Despite the good theoretical possibility, the role of IL-35 in asthma is still not well established. Wong et al. found significantly elevated IL-35 levels in patients with allergic asthma compared to those in nonallergic controls by evaluating plasma concentrations of IL-35 using an enzyme-linked immunosorbent assay (ELISA). Plasma concentrations of IL-35 were clearly positively correlated with the severity of asthmatic symptoms [27]. However, another study by Wang et al. reported completely opposite results suggesting instead that levels of circulating IL-35 are decreased in patients with asthma, and that decreased IL-35 levels increase the amount of IL-4-producing CD8^+^ T cells [28]. These contradictions in experimental results might be explained by the heterogeneity of airway inflammation in asthma and differences in the subtypes of asthma that were studied. Thus, additional studies with carefully phenotyped asthmatic patients are required to fully characterize the expression of IL-35 in the blood of asthmatic patients.

### 1.3. IL-35 in Experimental Models of Asthma

Increasing numbers of studies suggest that IL-35 can suppress airway inflammation and AHR in experimental models of asthma. Mice deficient in the IL-35 subunit EBI3 or p53 displayed higher degrees of airway inflammation, higher levels of IL-17, and sustained AHR compared to wild-type mice after the treatment with an OVA-lipopolysaccharide (LPS) sensitization and challenge protocol, suggesting that IL-35 could suppress AHR [13]. In the mouse OVA model of asthma, treatment with either adenovirus-mediated IL-35 [51] or recombinant IL-35 [52] suppressed both AHR and the degree of allergic inflammation. Although the OVA model of asthma is considered a “classic” model, it is also deemed to be too “artificial” and lacks the features of chronic airway inflammation. The effects of anti-IL-35 therapy has therefore also been studied in a model using the “real-life” allergen house dust mite (HDM). Mice with glucocorticoid-sensitive eosinophilic airway inflammation induced by a HDM allergen-specific memory/effector Th2 cell line that is treated with pVAX-IL-35 DNA via an intranasal route showed decreased allergen-specific airway inflammation, while intramuscular injection of pVAX-IL-35 suppressed levels of circulating allergen-specific and total IgE over an extended period of time [18]. Taken together, these studies suggest that IL-35 may be a new effective therapeutic agent for the treatment of allergic asthma.

Several different mechanisms have been suggested to mediate the effects of IL-35. IL-35 affects a wide range of different inflammatory cells. It is likely that IL-35 does not exert its anti-inflammatory effect via one single mechanism, but rather simultaneously affects a number of different inflammatory pathways.

#### 1.3.1. Th Cells

IL-35 induces differentiation of CD4^+^ effector T cells into a specific subset of Tregs called iTr35 cells. These cells express IL-35, but not Foxp3, TGF-*β*, or IL-10, and can suppress inflammation in animal models of experimental autoimmune encephalomyelitis (EAE) and IBD [1].

In vitro, IL-35 has different effects on effector T cells dependent on the inflammatory condition. Under acute inflammatory conditions such as acute infections, IL-35 induces expansion of both CD4^+^CD25^+^ and CD4^+^CD25^−^ T cells, but under conditions mimicking chronic infection/inflammation, IL-35 instead suppresses CD4^+^CD25^−^ effector cell proliferation and Th17 cell differentiation [11]. Spleen cells from mice deficient in the IL-35 subunit EBI3 display increased expressions of IL-17 as well as retinoid-related orphan receptor *γ*t (ROR*γ*t), which is the key transcription factor regulating Th17 cell differentiation [12].

Th17 levels are found to be elevated in the sputum of asthmatic patients and to correlate with their degree of airway hyperreactivity [41]. In mice, IL-35 effectively suppresses IL-17 production, while at the same time enhancing interferon-gamma synthesis, and thereby attenuates established CIA in vivo [7]. Treatment with exogenous IL-35 inhibits the development of inflammation in mice with CIA via suppression of Th1 and Th17 cells and production of IL-10 by CD25^−^CD39^+^CD4^+^ T cells [16]. In addition, IL-35 attenuates Th2-type airway inflammation induced by HDM allergen-specific memory/effector Th2 cells [18].

#### 1.3.2. Dendritic Cells

IL-35 has been shown to suppress dendritic cell (DC) migration to the lung and draining of mediastinal lymph nodes (mLNs) [52]. In a murine OVA model of asthma, IL-35 interrupts the conversion of the recruited monocytes to inflammatory DCs and at the same time attenuates the accumulation of migratory CD11b^+^CD103^−^ DCs in the mLNs and lungs. Mice that received IL-35 on days 1 and 7 during OVA sensitization had a decreased percentage and absolute number of CD11b^+^CD103^−^ DCs in the mLNs [52].

#### 1.3.3. Eosinophils and Neutrophils

Airway inflammation in the murine OVA model is predominately eosinophilic and accompanied by large amounts of Th2 cytokines, but small amounts of neutrophils and mononuclear cells can also be found. Intraperitoneal injection of IL-35 during sensitization significantly suppressed the inflammatory response, decreasing the number of infiltrating eosinophils and Th2 cytokine levels in BALF and lung tissue [52]. Moreover, in a model of airway inflammation induced by a HDM allergen-specific Th2 cell line, local administration of a plasmid that enhances IL-35 production significantly decreased the levels of eosinophilia, neutrophilia, total IgE, and the Th2 cytokine IL-4 [18]. Moreover, OVA-/LPS-induced neutrophilia and AHR in mice were inhibited by IL-35 via suppression of Th17 activity [13].

#### 1.3.4. B Cells

IL-35 is also known to affect antibody-secreting cells (ASCs), more specifically B cells. B cells boost immunity through the production of antibodies, but the subset of regulatory B cells (Bregs) can also suppress immunity through the production of IL-10, TGF-beta, and IL-35. Bregs, a set of ASCs that have been shown to play an interesting immunomodulatory role in allergic airway inflammation, are distinct [53]. They share a surface IgM^+^ CD138hi CD44hi CD69^+^ TACI^+^ C XCR4^+^ CD1dint TIM-1int MHC-II^+^ CD80^+^ CD86^+^ phenotype and express the transcription factors Blimp1 and IRF4. Mice with B cell-specific deficiency in IL-35 subunits survive longer following an infection with the *Salmonella* bacteria than control mice. This enhanced infection control was associated with increased macrophage activation and Th1 responses [53, 54].

## 2. Conclusion

The role of IL-35 in asthma is still not well established. Despite a growing body of evidence showing that IL-35 is an important regulator of inflammatory cell infiltration, activation, and clearance in experimental models, limited data are available regarding the levels and functions of IL-35 in human asthma, and published studies have provided contradicting results. These contradictions in experimental results might be explained by the heterogeneity of airway inflammation in asthma and differences in the subtypes of asthma studied. Thus, additional studies with carefully phenotyped asthmatic patients are required to fully characterize the expression of IL-35 in the blood of asthmatic patients.

Recent studies using a number of different murine models of asthma indicate that IL-35 exerts anti-inflammatory effects in vivo. It suppresses allergen-induced AHR and inflammation. Targeting the actions of IL-35 might therefore present a promising strategy for the development of effective therapeutic agents against asthma.

## Figures and Tables

**Table 1:** Serum IL-35 levels in different diseases and conditions.

Disease/condition	Method and assay	Level in blood	Comments
Inflammatory bowel disease (IBD)	ELISA (Bio-Swamp)	Crohn's disease: 454.17 ± 219.38 (ng/mL); ulcerative colitis (UC): 203.36 ± 38.21 (ng/mL); healthy control: 1788.96 ± 209.43 (ng/mL); mild UC: 358.26 ± 103.95 (ng/mL); moderate UC: 157.29 ± 15.89 (ng/mL); severe UC: 116.69 ± 14.48(ng/mL)	Serum IL-35 levels were decreased in IBD patients and also differed significantly between mild UC and moderate/severe UC [20]

Coronary artery diseases (CAD)	ELISA (Westtang Bio-tech, Shanghai, China)	Stable angina pectoris (SAP): 90.74 ± 34.22 (pg/mL); unstable angina pectoris (UAP): 72.20 ± 26.63 (pg/mL); acute myocardial infarction (AMI): 50.21 ± 24.69 (pg/mL); chest pain syndrome: 115.06 ± 32.27 (pg/mL)	Plasma IL-35 levels were significantly decreased in the SAP, UAP, and AMI groups compared to the chest pain syndrome group. Furthermore, IL-35 levels were moderately positively correlated with left ventricular ejection fraction (LVEF) in CAD patients [21]

Pancreatic ductal adenocarcinoma (PDAC)	ELISA (Cusabio Biotech, Wuhan, China)	PDAC: 134.53 ± 92.45 (pg/mL); healthy controls: 14.26 ± 6.56 (pg/mL)	PDAC patients had significantly increased level of circulating IL-35. Regulating the expression of IL-35 might therefore be a new possible target for the treatment of PDAC [22]

Non-small cell lung cancer (NSCLC)	ELISA (R&D Systems, Min neapolis, MN, USA)	NSCLC: 21.37 ± 11.55 (pg/mL); healthy controls: 10.09 ± 5.32(pg/mL)	Circulating IL-35 was significantly increased in NSCLC patients. IL-35 might therefore be a potential biomarker for predicting clinical outcome in NSCLC patients [23]

Acute pancreatitis	ELISA (BioLegend, San Diego, CA)	Acute pancreatitis: 5.25 ± 0.37 (ng/mL); healthy controls: 1.93 ± 0.16 (ng/mL); patients with severe attacks: 7.15 ± 0.48 (ng/mL); moderately severe attacks: 5.14 ± 0.49 (ng/mL); mild attacks: 3.69 ± 0.53 (ng/mL)	Increased serum IL-35 levels might relate to the inflammatory response in patients with acute pancreatitis. IL-35 might be a potential biomarker of acute pancreatitis [24]

Normal pregnancy	ELISA (Westtang Bio-tech, Shanghai, China)	Normal pregnancies: 333.6 (59.32–1391) (pg/mL); normal early pregnancy: 386.5 (64.37–1355) (pg/mL); nonpregnant women: 123.9 (8.763–471.7) (pg/mL); recurrent spontaneous abortion: 220.4 (4.951–702.0) (pg/mL)	Serum IL-35 levels increased in normal pregnancy and decreased in recurrent spontaneous abortion. IL-35 positively correlated with estrogen and alpha-fetoprotein (AFP) levels in early pregnancy. IL-35 might be an active player in the maintenance of a successful pregnancy [25]

Systemic sclerosis (SSc)	ELISA (USCN Life Sciences Inc., Hubei, China)	SSc: 83.9 (45.1–146.1) (pg/mL); healthy controls: 36.2 (17.2–49.4) (pg/mL)	IL-35 was overexpressed in skin, dermal fibroblasts, and serum of SSc patients. Increased serum IL-35 was associated with early inflammatory stages of SSc [26]

Asthma	ELISA (eBioscience Inc., CA, USA)	Asthmatics: 55.9 (6.6–419.0) (ng/mL); controls: 2.5 (0.1–16.1) (ng/mL)	Plasma concentrations of IL-35 were positively correlated with the severity of asthmatic symptoms [27]
	ELISA (BioLegend, San Diego, USA)	Asthmatics: 240 ± 120 (pg/mL); controls: 450 ± 190 (pg/mL)	Circulating IL-35 levels were decreased in patients with asthma. Decreased IL-35 levels increased the amount of IL-4-producing CD8^+^ T cells [28]

Data are expressed as mean ± SD or median (interquartile range).
